# Clinical and Research Activities at the CATANA Facility of INFN-LNS: From the Conventional Hadrontherapy to the Laser-Driven Approach

**DOI:** 10.3389/fonc.2017.00223

**Published:** 2017-09-19

**Authors:** Giuseppe A. P. Cirrone, Giacomo Cuttone, Luigi Raffaele, Vincenzo Salamone, Teresio Avitabile, Giuseppe Privitera, Corrado Spatola, Antonio G. Amico, Giuseppina Larosa, Renata Leanza, Daniele Margarone, Giuliana Milluzzo, Valeria Patti, Giada Petringa, Francesco Romano, Andrea Russo, Antonio Russo, Maria G. Sabini, Francesco Schillaci, Valentina Scuderi, Lucia M. Valastro

**Affiliations:** ^1^Laboratori Nazionali del Sud, Istituto Nazionale di Fisica Nucleare (INFN-LNS), Catania, Italy; ^2^Azienda Ospedaliero Universitaria Policlinico Vittorio Emanuele, Presidio Gaspare Rodolico, Catania, Italy; ^3^ELI-Beamlines Project, Institute of Physics ASCR, v.v.i. (FZU), Prague, Czechia; ^4^Medical Physics Section, Cannizzaro Hospital, Catania, Italy; ^5^National Physical Laboratory, Acoustic and Ionizing Radiation Division, Middlesex, United Kingdom

**Keywords:** proton therapy, dosimetry, clinical follow-up, Monte Carlo, laser-driven, ELIMED

## Abstract

The CATANA proton therapy center was the first Italian clinical facility making use of energetic (62 MeV) proton beams for the radioactive treatment of solid tumors. Since the date of the first patient treatment in 2002, 294 patients have been successful treated whose majority was affected by choroidal and iris melanomas. In this paper, we report on the current clinical and physical status of the CATANA facility describing the last dosimetric studies and reporting on the last patient follow-up results. The last part of the paper is dedicated to the description of the INFN-LNS ongoing activities on the realization of a beamline for the transport of laser-accelerated ion beams for future applications. The ELIMED (ELI-Beamlines MEDical and multidisciplinary applications) project is introduced and the main scientific aspects will be described.

## 1 Introduction

In developed countries, radiotherapy together with surgery is the most used approach for cancer therapy. The conventional and also most common form of radiotherapy make use of photons and electrons beams accelerated by linear accelerators. On the other hand, radiotherapy with hadron (protons and/or ions), in the last 10 years, is gaining more and more popularity thanks to its better physical and biological properties.

The use of energetic protons (energy sufficient to reach a tumor located in the human body) in medical applications was first suggested by Robert Wilson in 1946 (1) and in 1954 (2) the first patient was finally treated. Nowadays, according to the Particle Therapy Cooperative Group statistics (2), there are 58 active centers and 32 are under construction. Since first treatment, in 2015 about 154,000 patients have been treated with hadrontherapy. In Italy, the first hadrontherapy facility (see Section 2 for more details) started its operations in 2002. Since that time, two additional facilities have been developed and started their operation in Italy on the last decade: the CNAO foundation (3) where proton and carbon beams of 250 MeV and 450 AMeV, respectively, are available; and the proton therapy facility in Trento (web site: https://www.apss.tn.it/protonterapia).

Even if hadrontherapy, from many different reasons and aspects, is still a pioneering technique, nevertheless, its relevance in the clinical world and superiority with respect to the conventional radiation is evident for many clinical cases. It represents the election therapy in most of the choroidal and iris melanomas occurrences. In the case of the pediatric medulloblastoma, where the whole brain and spinal chord is irradiated, proton therapy greatly reduces the dose in the healthy tissue and sensibly reduces the associated risks of secondary tumor occurrence. In the breast cancer treatment, finally, is becoming more and more evident that, the use of protons, produces evident advantages like the reduction of the occurrence of lung secondary tumors and coronary diseases. The reader is suggested to read the excellent following list of publications reporting the current status of hadrontherapy and its principal advantages and drawbacks (4–13): and references therein.

Despite the evident advantage over conventional radiotherapy, the spread is limited by the high costs and complexity of the facilities. In this framework, the authors in Ref. (4, 12) clearly state that further in the future we will probably see the “*the first proton single room facility based on the illumination of a thin target with powerful (10^18^–10^20^ Wcm^−2^) and short (30 fs to 50 fs) laser pulse*.” At moment, the major challenges in laser-driven based radiotherapy is the development of a well-controlled, reliable, energetic ion beams of very high quality able to meet the medical requirements adopted in the clinical routine (see Section 5.3).

## 2 Catana, the Italian Eye Proton Therapy Facility

The *CATANA* (Centro di AdroTerapia Applicazioni Nucleari Avanzate) facility, built thanks to the collaboration between INFN-LNS and Public Health Policlinic named AOU-Vittorio Emanuele of Catania (I), is operational since 2002 and successfully treated more than 300 patients. The facility is dedicated to the radiation treatment of ocular melanomas with the 62 MeV proton beams accelerated by the INFN-LNS superconducting cyclotron. The most frequent neoplasia treated with proton beams is the uveal melanoma, followed by other eye diseases like choroidal metastases, conjunctival tumors, and eyelid tumors (14, 15). The CATANA facility is based on a passive transport system of a 62-MeV proton beam. The proton maximal range, at the irradiation point, is about 30 mm, ideal for the treatment of eye tumors. The necessary maximum range and energy modulation are achieved by means of a set of Perspex absorbers, variable in thickness, and modulator wheels.

### 2.1 Main Characteristics of the Beamline

**Figure 1:**
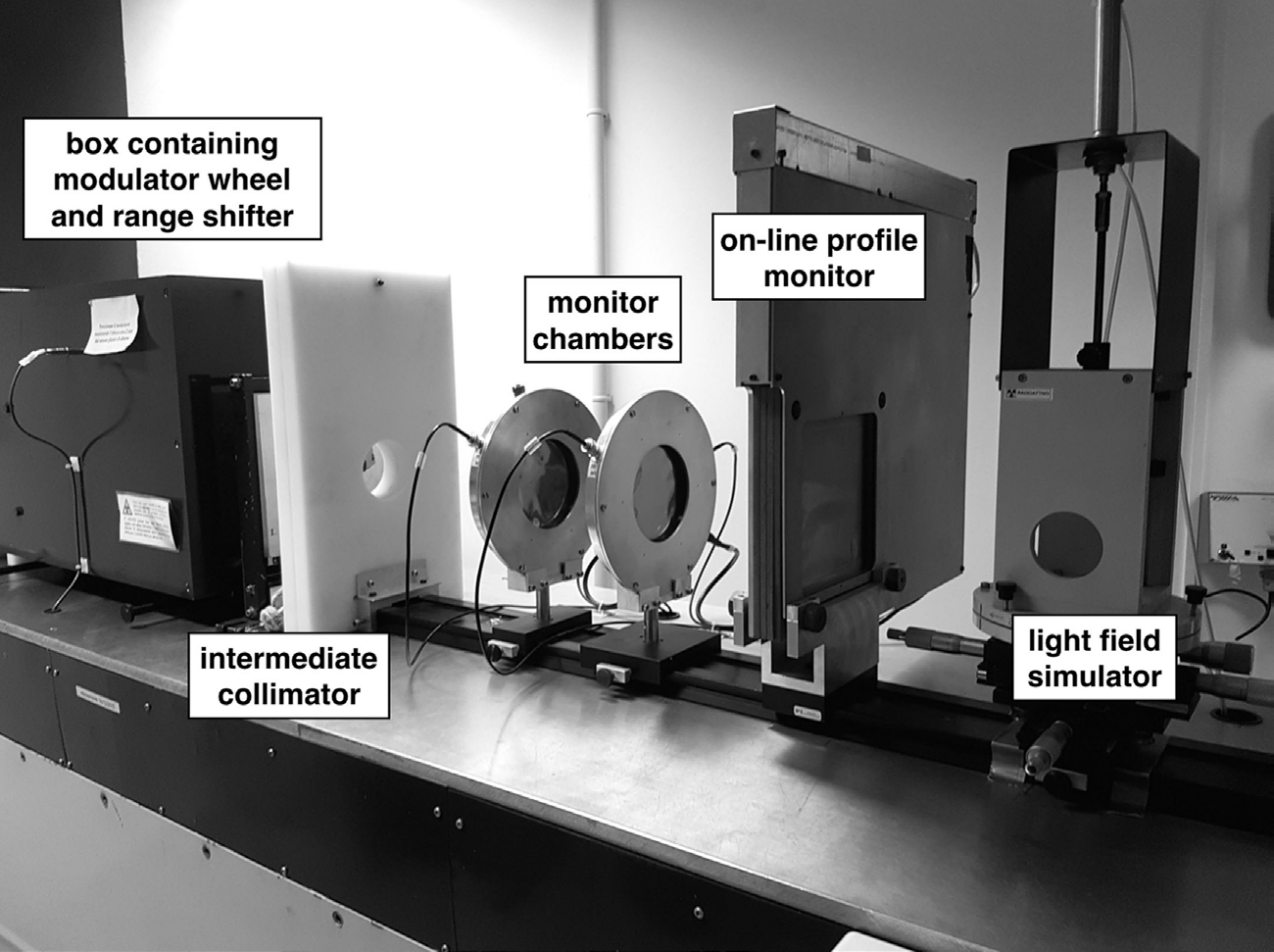
Central section of the CATANA proton therapy beamline with some transport (collimators, modulators, and range shifters) and diagnostic (monitor chambers, on-line profile monitoring, and field simulator).

**Figure 2:**
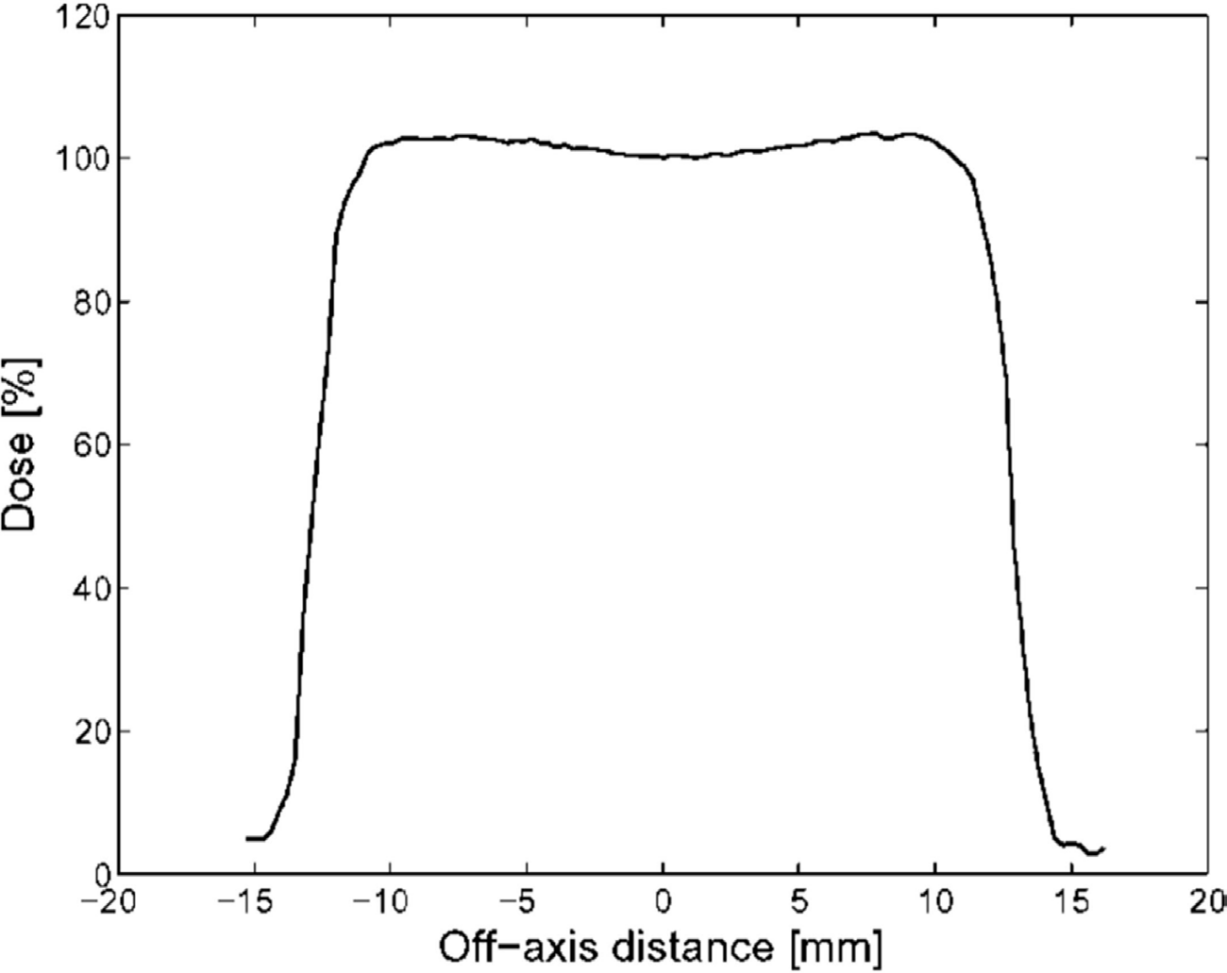
Experimental lateral dose distribution acquired with a silicon diode. This picture reproduced with the permission of the authors.

The CATANA beamline has been developed at INFN-LNS of Catania (Figure 1). Accelerated protons exit in air through 50 µm Kapton window. Upstream the exit window, a first thin (15 µm) tantalum scattering foil is placed in vacuum: it performs a first broadening of the beam. After the Kapton window, in air, a second thicker (25 µm) tantalum foil, equipped with a brass stopper of 4 mm in diameter, is used to perform the second beam scattering. This double foil scattering system is designed to obtain an optimal homogeneity of the final proton beam, in terms of lateral dose distribution contemporary minimizing the energy losses. A typical experimental transversal dose distribution for the 62-MeV clinical proton beam is shown in Figure 2. Reported data are acquired in water with a Hi-pSi diode (0.6 mm detector diameter) at 12 mm water-equivalent in depth, corresponding to the middle of a Spread Out Bragg Peak (SOBP). Range shifter and range modulator are positioned downstream the scattering system. The radiation field is simulated using a diffused light field. Two transmission monitor ionization chambers, providing the on-line control of the dose delivered to the patient represent the key elements of the patient dosimetry system (16–18).

## 3 Beam Dosimetry and Monitoring

The procedures and methods to perform the absolute and relative estimation of the released dose constitute a key point in the life of an hadrontherapy facility. Because of steep dose distal and lateral gradients, detectors with high spatial resolution, energy, and dose rate independent have to be used.

In the case of monoenergetic and modulated proton beams, only plane-parallel chambers are recommended for measuring depth–dose distributions; ion chambers must fully satisfy IAEA requirements (19) as for electrode separation (*h* < 2 mm), guard ring width (≥1.*5 h*), cavity diameter to cavity height (≥5), and bias voltage. The PTW TM34045 *Advanced Markus*, parallel-plate ion chamber (*V* = 0.02 cm^3^) was adopted as reference for depth–dose measurements at CATANA proton therapy facility. Dose measurements are carried out in a water phantom where the chamber is moved with a scanning resolution of the order of 0.1 mm.

**Figure 3:**
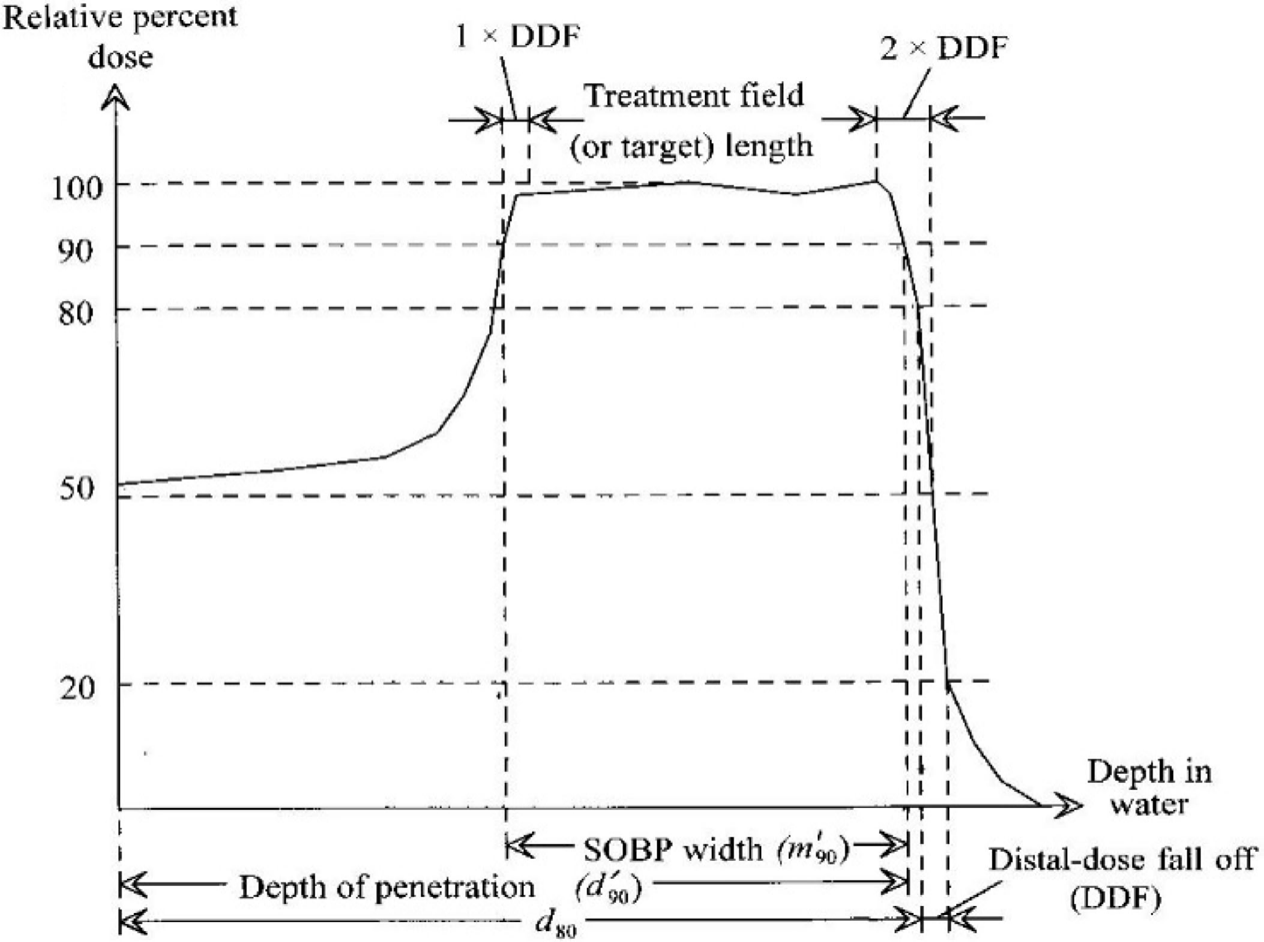
Parameters to evaluate modulated proton beam.

For modulated proton beams, a set of physical parameters have to be measured, strictly connected to the needed clinical requirements (Figure 3):

1. The beam range, referred as the 90% distal point of the peak;
2. The extension of the SOBP, defined as the distance of 95% dose points, proximal, and distal;
3. The reference depth (*z_ref_*);
4. The beam quality (Q), measured as the value of the residual range, defined as *R_res_* = *R_p_* − *z_ref_*, where *R_p_* is the depth at which the absorbed dose beyond the SOBP falls to 10%.
5. The longitudinal homogeneity, defined as $\left(\frac{D_{\text{max}}}{D_{\text{min}}}\right)\times 100\%$ within the SOBP.

At the CATANA proton therapy facility, different detectors (Diamonds, p-type silicon diodes) were tested to be used for the characterization on modulated proton beams as an alternative to the Advanced Markus chamber. The PTW Dosimetry Diode PR Type 60020 is a p-type silicon diode detector with physical dimensions of 7 mm diameter, 45.5 mm length, and an extremely small sensitive volume of 0.02 mm^3^ (20).

**Figure 4:**
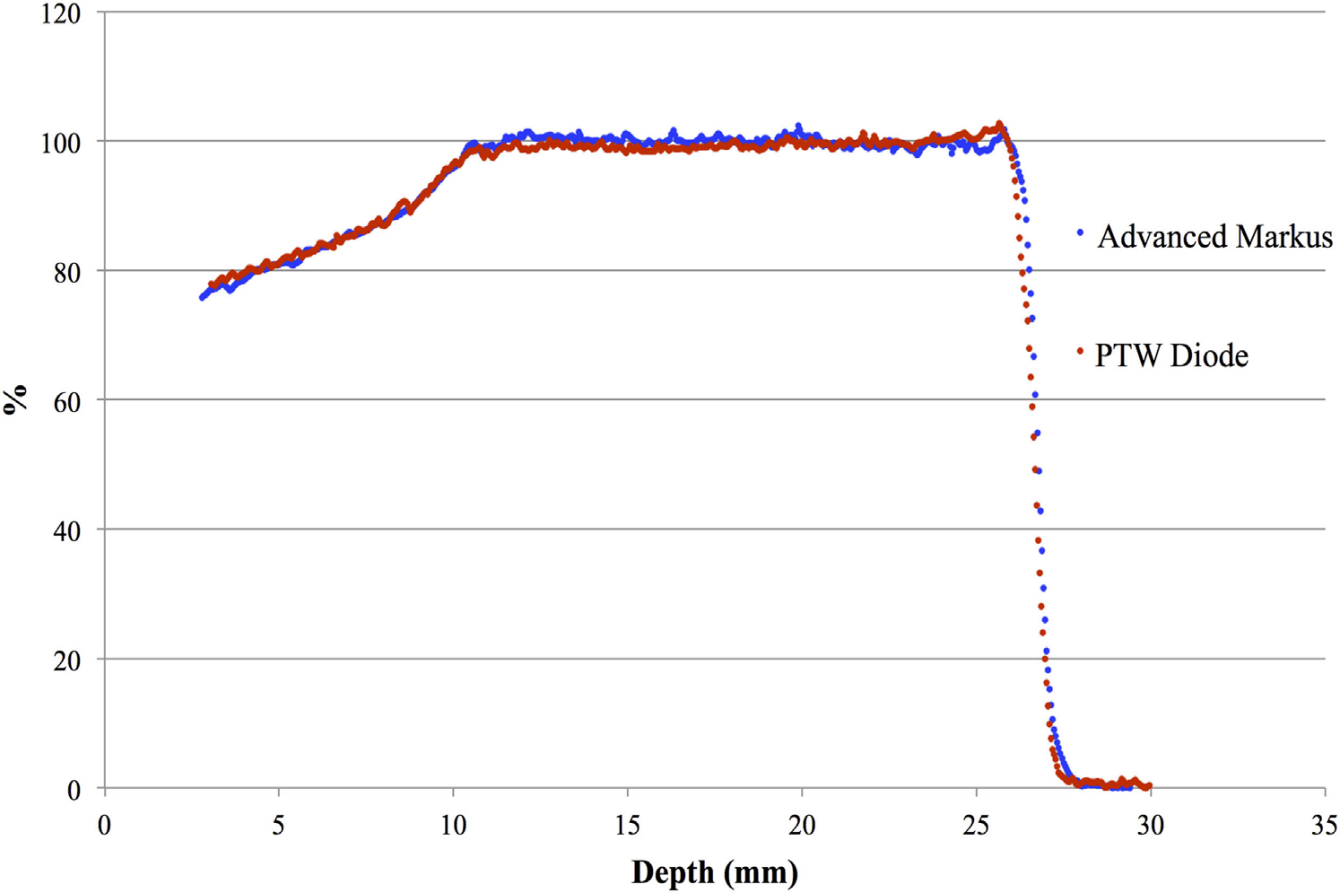
Central axis depth–dose distribution of a modulated clinical proton beam measured by the PTW diode PR 60020 and the Advanced Markus Chamber. This picture reproduced with the permission of the authors.

All measurements with the PTW diode were performed with the detector axis parallel to the beam axis (axial orientation), as recommended by the manufacturer for application in clinical proton beams. No polarization field is used for the silicon diode and a PTW Unidos Type E electrometer is adopted for the current measure. Figure 4 reports the depth–dose distribution of a modulated proton beam acquired by the PTW diode PR 60020 and the Advanced Markus Chamber; as recommended, data were normalized to the middle of SOBP, i.e., at reference depth (*z_ref_*).

The dose distribution physical parameters of the clinical beam are reported in Table 1 where the results obtained from the PTW diode are compared with the reference ones obtained with the Advanced Markus chamber. Negligible differences, all within the experimental uncertainties related to detector positioning and determination of effective point of measurements, are observed.

**Table 1:** Comparison of the parameters measured by the PTW diode PR 60020 and the Advanced Markus Chamber.

	Advanced Markus [mm]	PTW diode [mm]	Difference [mm]
95% Prossimal	9.772	9.706	0.066
95% Distal	26.203	26.098	0.105
Range (90%)	26.380	26.188	0.193
Penumbra (80–20%)	0.488	0.629	0.140
SOBP width (95−95%)	16.431	16.391	0.039
Penumbra (90–10%)	0.799	0.898	0.099

The lateral dose profiles of a passively scattered beam are characterized in terms of:

1. Field size (dimension of the transversal dose distribution profile at the level of *W*50%).
2. Lateral penumbra (LP, *d*80–20%).
3. Flatness and symmetry.

GafChromic EBT3 film is the reference detector for measurement of lateral dose profiles, because of a nearly water-equivalent effective atomic number (*Z_eff_*(EBT) = 6.98 compared to *Z_eff_*(*water*) = 7.3) and sub-mm spatial resolution (up to 100 µm), when read out by conventional flatbed scanners. Irradiated films are digitized in transmission mode 24 h after irradiation. Scanning is performed using the flatbed scanner EPSON Expression 10000XL, and the red channel of 48 bit RGB images is extracted and saved. EBT3 calibration (21) is carried out on the horizontal beamline of the CATANA facility. Several strips 3 cm × 3 cm are irradiated in a Solid Water phantom at 1 mm depth in the entrance plateau of the Bragg curve, corresponding to a *residual range* of about 30 mm; the reference 25 mm diameter circular collimator is used for calibration. Films are irradiated to a proton dose in the range of 0.25–4 Gy at a dose rate of 15 Gy/min, corresponding to the eye clinical dose rate. Calibration curves for 62 MeV protons is well fitted by a third order polynomial and is in agreement with the curve for 6 MV photon beams, indicating a nearly water equivalence of the EBT3 film.

The response in dose of EBT3 film was found to be energy independent (≤2%) in the energy range of eye proton therapy. This range corresponds to residual ranges beyond the irradiation depth that stays in the interval between 6 mm (25 MeV) and 29 mm (58 MeV). For these reasons, the EBT3 films can be used for dosimetric verification (1D, 2D distributions) of standard circular collimators as well as of irregularly shaped patient collimators with very small lateral extension (Table 2).

**Table 2:** Field parameters measured with the EBT3 film along the X- and Y-axis.

	Field size [mm]	W_95%_ [mm]	Penumbra [mm]	Homogeneity [%]	Symmetry [%]
Axis Y	20.82	16.64	1.6	2.8	104.3
Axis Z	13.25	9.89	1.5	0.9	101.5

**Figure 5:**
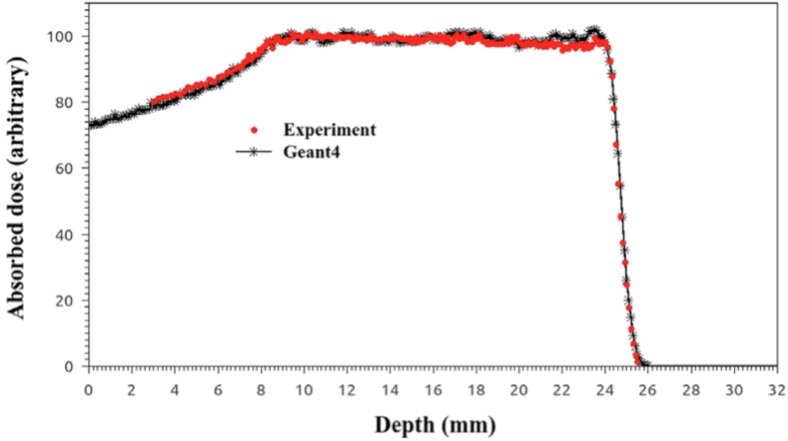
Comparison between simulated and experimental depth–dose distributions with a modulator realized using the new approach.

Both cylindrical and plane-parallel chambers, calibrated in terms of absorbed dose to water at the reference quality *Q*_0_ $\left(N_{D,w,Q_{0}}\right)$, are recommended for use as reference instruments for the Users proton beam calibration. For low proton energies, as for eye proton therapy, with SOBP smaller than 2 cm, plane-parallel ion chambers must be used. In modulated clinical proton beams, the monitor chambers are calibrated (in terms of cGy/U.M.) by a dose measurement in the middle of SOBP according with the recommendations of TRS-398 (19). The absolute value of the absorbed dose in water, at the calibration depth in a proton beam of quality Q, is calculated according to the following expression:

(1) $$D_{w,Q}={\text{M}}_{Q}\times N_{D,w,Q_{0}}\times k_{Q,Q_{0}}$$

where *M_Q_* is the reading of the dosimeter at the reference position; $N_{D,w,Q_{0}}$ is the calibration factor in terms of absorbed dose to water for the dosimeter at the reference quality *Q*_0_; The factor $k_{Q,Q_{0}}$ is a chamber-specific factor that accounts for the difference between the reference quality (*Q*_0_) and the user proton beam quality; the reference beam (*Q*_0_) is generally the ^60^Co. The Classic PTW TM23343 parallel-plate Markus chamber (V = 0.055 cm^3^) has been adopted at CATANA proton therapy facility as reference dosimeter (22). Beam calibration is provided for the reference circular collimator 25 mm in diameter. As for all passive systems, a single calibration has to be performed for each individual treatment field, because of the strong dependence of the beam calibration on range shifter thickness and SOBP width (see Figure 5) (22, 23). The variation in the Output Factor $\left(\text{OF}=\frac{\text{cGy}}{\text{Monitor Units}}\right)$ with decreasing beam area has been measured at the beamline commissioning, for the same monitor unit setting, by radiochromic films; the experimental results were normalized to the reference collimator output. We found that the beam output factor decreases by less than 3% over a range of field area from 490 mm^2^ (reference collimator) to the smallest clinical used (about 50 mm^2^).

## 4 Monte Carlo Simulations

Use of the Monte Carlo simulation is of extreme importance in Hadrontherapy. Monte Carlo is, in fact, the most precise approach for the calculation of dose deposition in human tissues being able to exactly reproduce the anatomical structures and the complex particle beams involved in an hadrontherapy treatment.

### 4.1 Monte Carlo Simulation of the CATANA Beamline

**Figure 6:**
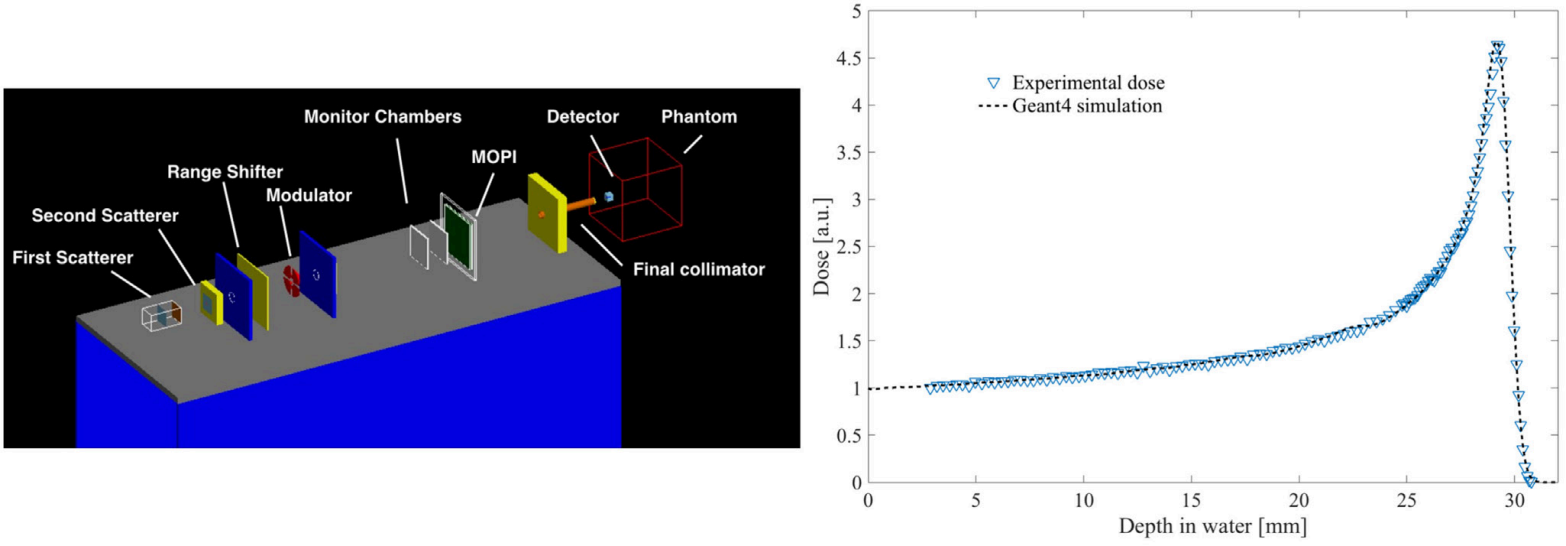
Screenshot from the Hadrontherapy application with the complete simulation of the CATANA proton therapy beamline (left); comparison between and experimental and simulated pristine Bragg peaks (right).

The CATANA beamline has been simulated in details using the Monte Carlo code Geant4 (24, 25). It is a toolkit for the simulation of particle tracking in the matter, written in C++, and developed by an international collaboration composed of more than 100 of Researchers coming from the most important Institutes worldwide. Initially developed for the simulation for high-energy physics experiments, it is now widely used in several fields, as space and medical applications (26). More than 10 years ago, we developed a free and open source application that simulates the CATANA transport beamline, named Hadrontherapy (Figure 6 (left)), currently available inside the public release of the Geant4 code (27). In the last years, the application has been sensibly improved, introducing several modules dedicated to the study of different aspects. It is now configured as a general-purposes example that aims to study issues related to hadrontherapy with protons and light ion beams. Hadrontherapy allows the simulation, *via* simple macro commands, of the whole transport beamline including all the necessary transport elements. The application has been extensively validated against experimental data; as an example, depth–dose distributions in water for 62 MeV proton beams obtained with the Geant4 Hadrontherapy example are compared in Figure 6 (right) with the experimental ones. Once validated, the application has been used also in the clinical practice, to support the patient treatments especially for specific cases where complications due to the anatomical configuration may introduce uncertainties in the treatment plan. In this concern, depth–dose distributions for the clinical cases have to be considered (SOBPs) and they are obtained with a modulator wheel in PMMA. A dedicated module has been developed for the simulation of this element. Recently, it has been deeply revised to provide the Users’ with an easier tool for changing the modulation region according to the different longitudinal target sizes. In the following subsection, more details are given, showing also some benchmarks with experimental data.

### 4.2 New Approach for the Simulation of a Modulator Wheel

With the developed new modulator class, very specific modulators can be realized. The data in the input file are the number of modulator steps (including air gap), the thickness of each step (zero for air gap), and the absolute or relative weight of each step. A debugging activity has been carried out to fix some issues related to the obtained depth–dose distributions and to predict in a realistic way the experimental data. As a final result, in Figure 5, it is shown, as example, a comparison of the experimental SOBP and the one obtained with the Geant4 simulation using the new approach for the modulator design, where the good agreement between the two distributions is clearly visible.

### 4.3 Average LET Distributions

**Figure 7:**
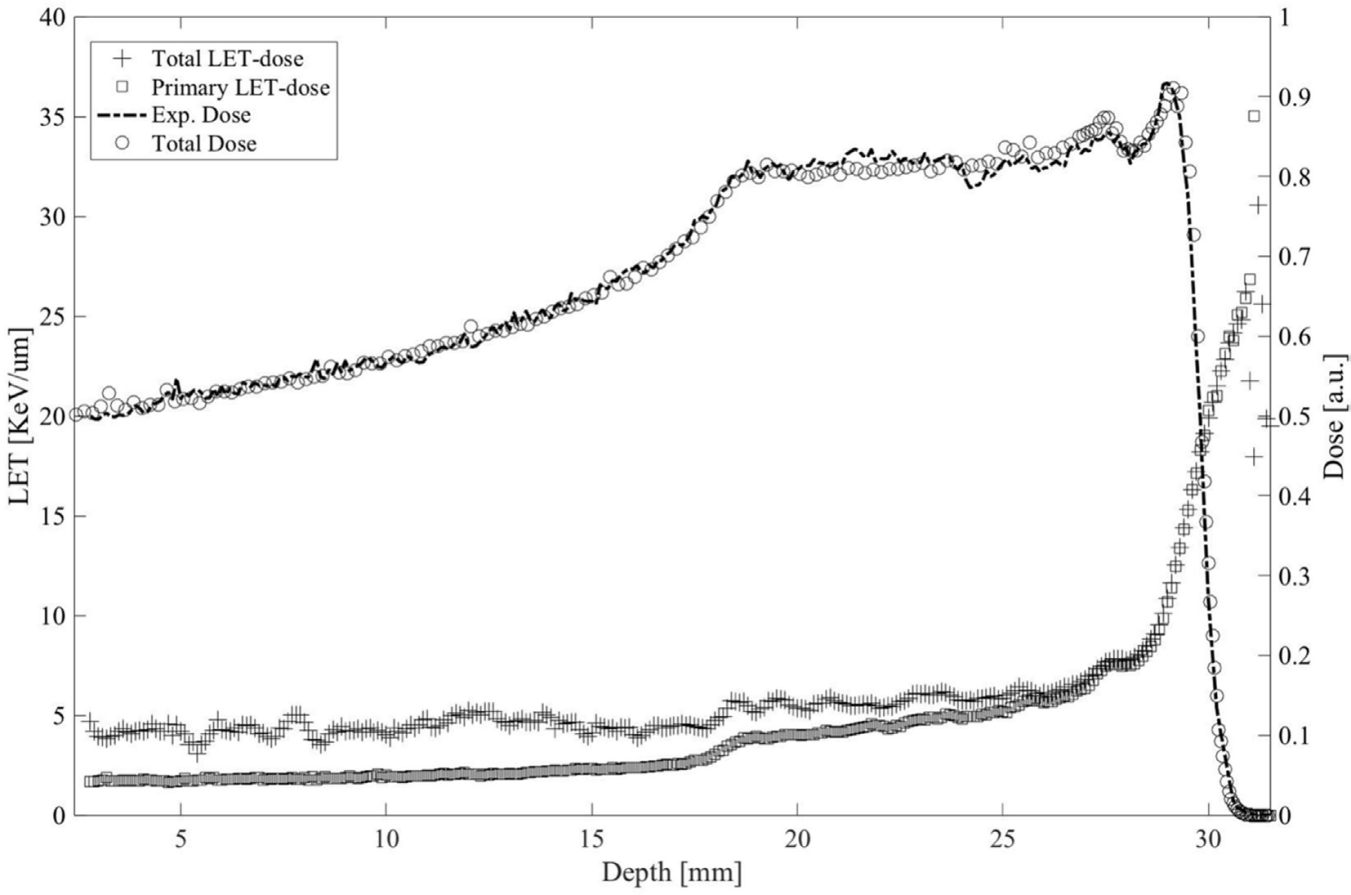
Averaged LET-dose distributions calculated for only primary ion (square) and considering the contribute of the generated secondaries (cross) for a clinical spread-out Bragg proton peak calculated in water using the Hadrontherapy Geant4 simulation. Experimental and simulated SOBPs are also shown.

As already mentioned, a huge research activity has been carried out in these years in parallel to the clinical activity related to the proton therapy treatments. In particular, thanks to the collaborations with other INFN Sections in Italy and also different Institutions in Europe, several radiobiology experiments have been performed to study the biological effects induced on tumor and healthy cells respect to different irradiation conditions. One of the most studied parameters on this concern is the dependence of the biological damage on the radiation quality, typically quantified by means of the average Linear Energy Transfer (LET). In particular, the dose-averaged LET is of great interest in radiobiology because, according to its definition, it takes into account also the different energy contribution of the primary beam, correctly weighting for the deposited dose at a specific depth. We developed a dedicated module inside the Hadrontherapy application to study the LET-dose distributions in configurations that are of interest for radiobiology experiments (28). In particular, we proposed and tested a tool allowing to compute the dose-averaged LET considering in the computation also the contribution due to secondary particles produced for nuclear interactions. We found that a non-negligible difference there is in the average LET in the entrance channel, if the secondary contribution is also taken into account. Indeed, an LET three time higher has been obtained in this case, respect to the one retrieved when only primary incident protons at 62 MeV are considered, which is about 1 keV/μm (Figure 7). Similar results have been obtained also for carbon ion beams, even though the effects of secondary particle contribution is quite different, and the same computations can be done for each kind of incident ion. Recently, we have developed a new algorithm that makes the LET calculation completely independent from simulation transport parameters. It is based on the using of a specific function implemented inside the Geant4 kernel, belonging to the class G4EMCalculator, which converts the energy of charged particles to unrestricted LET directly. This module is now included in the public version of the Hadrontherapy advanced example, since the last release of the Geant4 code, so that all the interested Users’ can download and use it.

## 5 Clinical Activity: Treatment Procedures and Results

### 5.1 Treatment Procedure and Patient Positioning

The knowledge of the tumor exact position is essential for eye proton therapy. To achieve this, radioopaque Tantalum clips, implanted around the lesion on the outer sclera, are used as reference points during the planning and irradiation phase. The surgeon also defined the tumor position and measurements as transverse and longitudinal base diameters, elevation or height, distance to the optic disk and to the macula. The final result of this procedure is a precise virtual reconstruction of the tumor and healthy tissue around it. Eye and tumor are reconstructed by the EYEPLAN treatment planning system that provides a correct proton dose distributions and eye position during the treatment.

Before the treatment starts, the patient is immobilized. First of all, a fixation device is made by means of a customized thermoplastic mask and bite block, with the patient in a seated position. It is required the patient to gaze a light point and two orthogonal X-ray images (axial and lateral) are acquired. The radiographic system, based on two flat panels HAMAMATSU model C7921CA-02, is able to identify the eye position at the isocenter point through a comparison between the radiographic images and simulated reconstructions obtained by using EYEPLAN. The patient positioning chair is then moved to remove all misalignments. A measurement of the eyelid thickness and slope is also carried out to complete the planning procedures. The EYEPLAN software schematically displays a model of the patient eye (including the other anatomical parts such as lens, optic nerve, and fovea), and it provides a finally drawn of the tumor by means of the specified measurements and positions. The planned isodose levels for 90, 50, and 20% of the prescribed dose and the DVH of the tumor and the organ at risk are reported by the treatment planning system. Before the treatment is also verified the patient position because if eyelids cannot be retracted completely outside the irradiation field, they have to be included in the treatment plan, as well. A CCD camera is used to continuously verify the eye position during the irradiation. The treatment time is between 30 and 60 s.

### 5.2 Last Clinical Results

**Figure 8:**
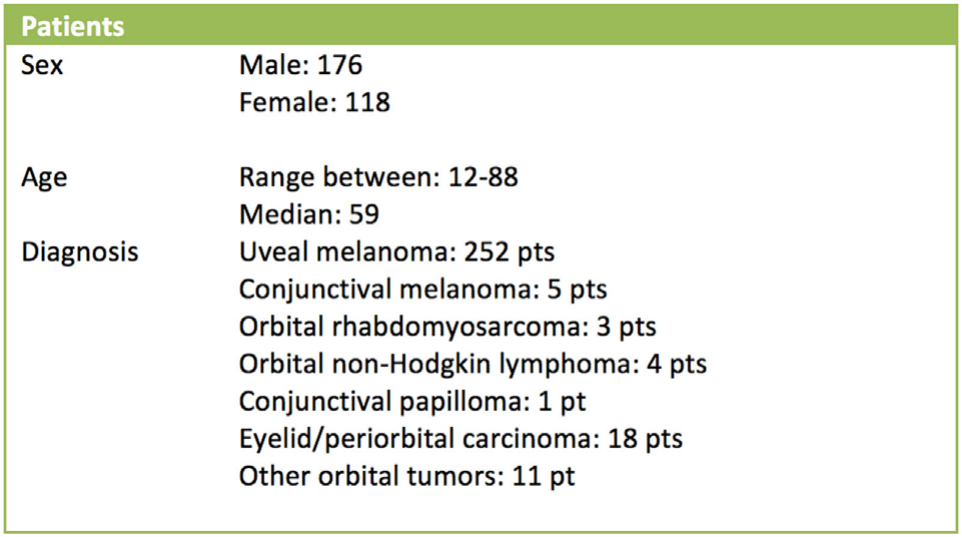
Characteristics of the treated patients.

During the first 14 years of clinical activity, more than 300 patients have been treated at CATANA facility. Uveal melanoma has been the most frequent treated tumor, accounting for 252 treatments. Some other neoplasia has been treated by means of proton beams: conjunctival melanoma 5 patients, orbital rhabdomyosarcoma 3 patients, orbital non-Hodgkin lymphoma 4 patients, conjunctival papilloma 1 patient, eyelid and periorbital tissue carcinoma 18 patients, choroidal metastases, and other orbital tumors 11 patients (Figure 8).

Proton beam dose applied was the same for all melanoma patients: 60 Gy [RBE] are delivered in a hypofractionation regimen, with 4 fractions on 4 consecutive days. An RBE of 1.1 is applied for the whole physical dose distribution (SOBP). For other tumors, a lower total dose was given, ranged between 30 and 48 Gy [RBE], with the same hypofractionated regimen.

Proton beam radiation therapy (PBRT) is considered as a gold standard in the eye-conservative treatment of uveal melanoma, the most frequent ocular tumor in the adult, having demonstrated a tumor control rate and an overall survival rate comparable to those of enucleation trials (29–31). There are no available data from randomized trials for the application of proton beams to the treatment of other histologies, but only anecdotal data from case studies or single institution experiences. Then, inspired by the results of the proton beams in the treatment of uveal melanoma, so also for the other tumors of the orbital and periorbital region a conservative approach with proton therapy has been applied for 42 patients, for which there was no other therapeutic options.

**Figure 9:**
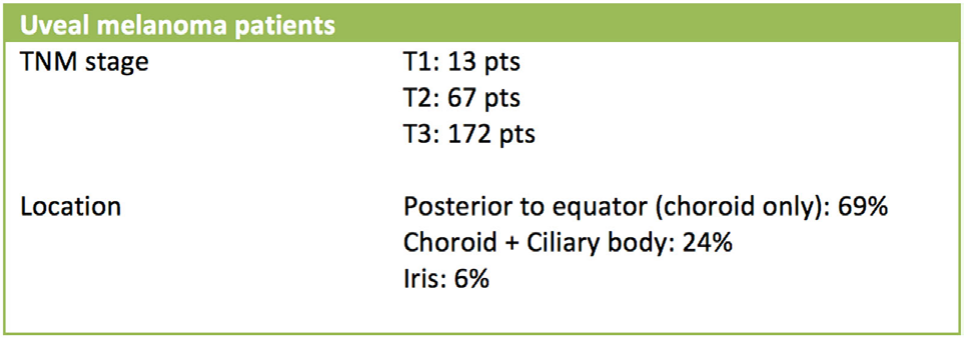
Stage and location distribution (uveal melamona).

Follow-up is available for all patients treated. It ranges from few months to 14 years, so the data can be considered mature for statistical considerations. Taking in account patients affected by uveal melanoma, according to the TNM-AJCC staging system (VII edition, 2010), they were classified as follow: T1 for 13 patients, T2 for 67 patients, and T3 for 172 patients (252 patients in total) (Figure 9).

**Figure 10:**
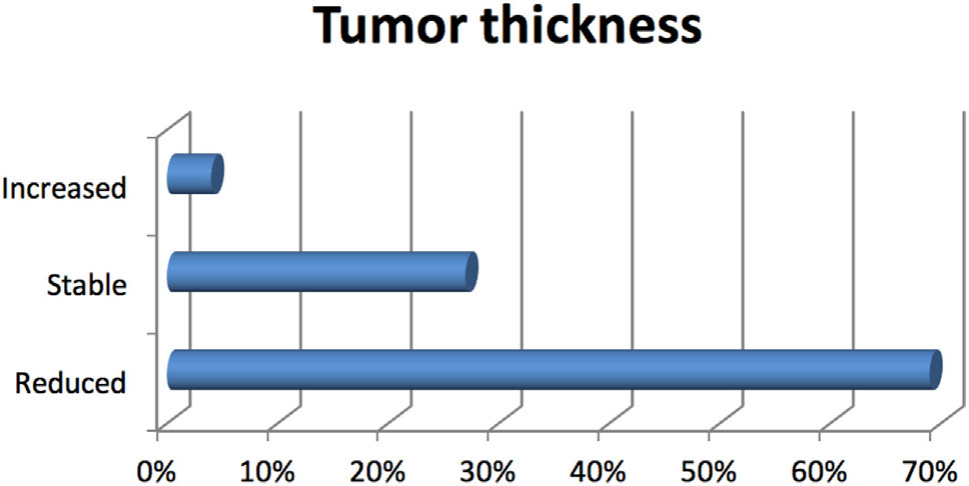
Local control response by tumor thickness.

**Figure 11:**
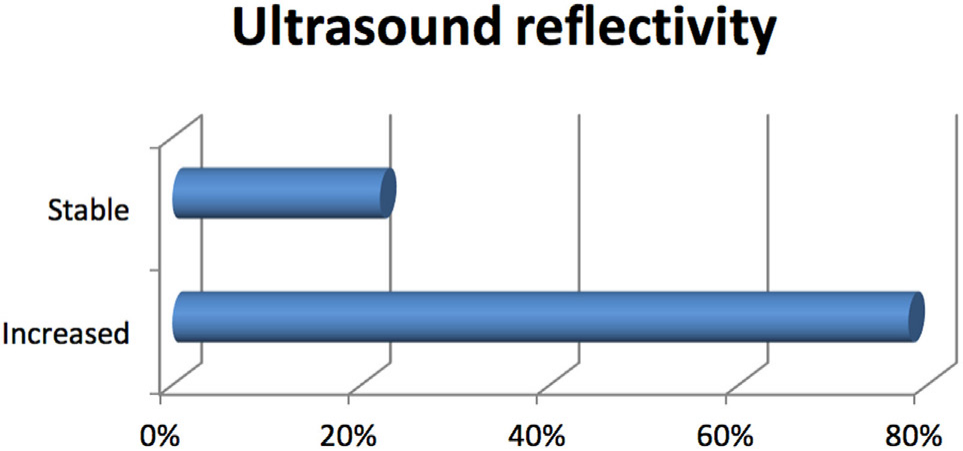
Local control response by ultrasound reflectivity.

The majority of uveal melanomas were located posteriorly to the eye equator (75%), in close vicinity to the vision OARs, optic disk, and macula. A tumor local control is the primary endpoint of ocular proton therapy: it is defined as a dimensional reduction or stabilization of the elevation of uveal tumor from the retinal surface. A surrogate of dimensional evaluation is the increasing tumor ultrasound reflectivity, especially in the case of thickness stabilization. According to these definitions, our patients have obtained a tumor control in 96% of cases (Figures 10 and 11). A secondary endpoint of ocular tumor proton therapy, as a conservative approach alternative to eye enucleation, is the maintenance of the eye (29). Independently from the tumor control and from the residual visual function, 229 patients (90%) have maintained their eye. The major cause of secondary radiation-induced eye enucleation was neovascular glaucoma, in many cases occurring for big tumor volumes, conditioning an irradiation of large retinal surface. The time of enucleation ranged between 1.5, and 4 years from the completion of PBRT. In the group of patients with other histologies, a secondary enucleation was required. Another goal of ocular proton therapy is the maintenance of a functional eye, with an acceptable visual acuity. This is, of course, only possible in patients with a tumor located, at diagnosis, away from optic disk or macula, and with a visual function not yet compromised by other eye diseases. Taking into account these premises, in our series, a functional eye was maintained in about 40% of patients (18, 32, 33).

Ocular proton therapy is the treatment of choice in most ocular and orbital tumors, due to the high conformal treatment isodoses and the ability to spare the healthy surrounding tissues better than photon-beam treatment techniques. Despite this, due to the local extension and location of disease onset, the development of radiation-induced damages is often unavoidable. In our experience, radiation retinopathy of various degrees was seen in 22% of patients, radiation-induced cataract was detected in 35% of patients, and neovascular glaucoma developed in 18% of patients. Cause-specific survival was 92%, since 18 patients affected by uveal melanoma and 3 affected by other tumors died from metastatic disease. Ocular melanoma, both uveal and conjunctival, has a strong tendency to metastasize, especially in the liver, after many years from diagnosis, regardless of the local control of the primary tumor [H].

### 5.3 Potential Medical Applications of Laser-Driven Beams: The ELIMED Beamline at ELI-Beamlines

The acceleration of charged particle *via* ultra-intense and ultra-short laser pulses has gathered a strong interest in the scientific community in the past years, and it represents nowadays one of the most challenging topics in the relativistic laser–plasma interaction research. Indeed, it could represent a new path in particle acceleration and open new perspectives in multidisciplinary fields. Among many scenarios, one of the most interesting idea driving recent research activities consists in setting up high intensity laser–target interaction experiments to accelerate ions for medical applications, with main motivation of reducing cost and size of acceleration, currently associated with big and complex acceleration facilities (34, 35).

Indeed, a development of more compact laser-based therapy centers could lead to a widespread availability of high-energy proton and carbon ion beams providing hadron therapy to a broader range of patients (34, 35).

However, to assume a realistic scenario where laser-accelerated particle beams are used for medical applications, several scientific and technological questions have to be answered and requirements to be fulfilled. Furthermore, the properties of laser-driven proton bunches significantly differ from those available at conventional accelerators, both in terms of pulse duration and peak dose rate. Thus, many scientific and technical challenges must be solved, first to demonstrate the feasibility of unique applications with laser-driven ion beams, and second to perform reliable and accurate physical and dosimetric characterization of such non-conventional beams, before starting any medical research and application. Different acceleration regimes have been experimentally investigated in the intensity range 10^18^–10^21^ W/cm^2^ in the so-called Target Normal Sheath Acceleration (TNSA) regime (36–38). Acceleration through this mechanism employs relatively thin foils (about 1 µm), which are irradiated by an intense laser pulse (of typical duration from 30 fs to 1 ps). At peak intensities of the order of 10^18^ W/cm^2^ hot electrons are generated in the laser–target interaction whose energy spectrum is strictly related to the laser intensity itself. The average energy of the electrons is typically of MeV order, e.g., their collisional range is much larger than the foil thickness. Hence, they can propagate to the target rear and can generate very high space-charge fields able to accelerate the protons contained in the target. The induced electric fields, in fact, are of the order of several teravolts per meter and, therefore, they can ionize atoms and rapidly accelerate ions normal to the initially unperturbed surface. Typical TNSA ion distribution shows a broad energy spread, exceeding 100%, much larger compared to the 0.1–1% energy spread typical of ion beams delivered by conventional accelerators, a wide angular distribution with an half-angle approaching 30° which is very different from the typical parallel beam accelerated by the conventional machine and a very high intensities per pulse, i.e., up to 10^10^–10^12^ particles per bunch, as well as a very short temporal profile (ps) compared to 10^7^–10^10^ particles/s of conventional clinical proton beams. Moreover, the cutoff energy value can be likely considered as a spectrum feature still strongly dependent on the shot-to-shot reproducibility and stability and up to now, the maximum proton energy obtained with a solid target in the TNSA regime is about 85 MeV (39). In the last years, a significant amount of theoretical and experimental attention has been dedicated to explore other acceleration schemes that are expected to appear for intensity higher than 10^21^ W/cm^2^ and ultra thin foils (40–44). The study on the optimization of the laser-driven source features has been coupled to the experimental investigations carried out on target nano-structures (45, 46) and recently also very innovative cryogenic technologies (47). Different types of structured target have been recently developed and tested aiming to improve the characteristics of the optical-accelerated beam at the source.

**Figure 12:**
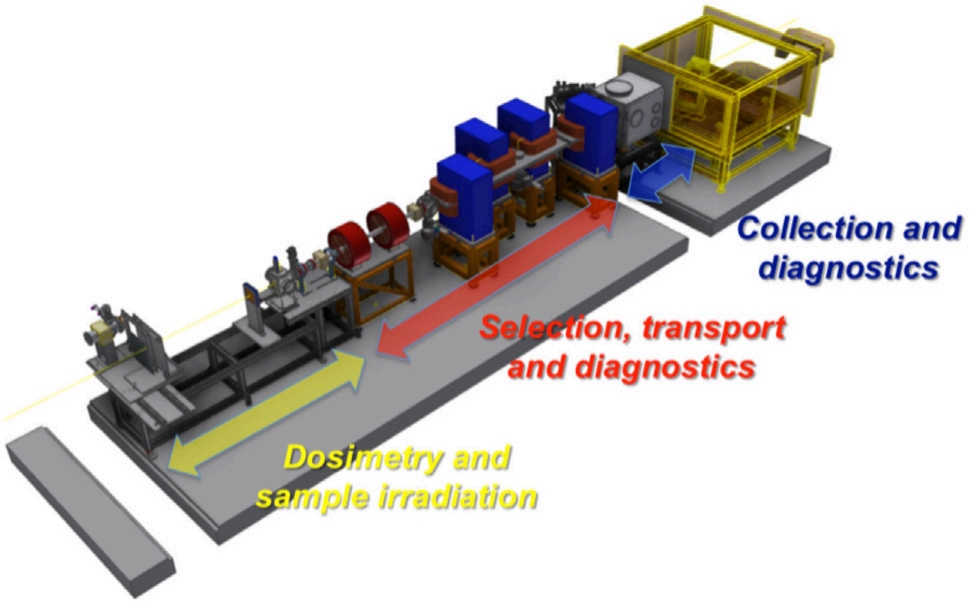
Layout of the ELIMED beamline with the three different sections. This picture reproduced with the permission of the authors.

These results are particularly promising along the pathway for achieving laser-driven ion beams matching the parameters required for different multidisciplinary applications, including the medical ones. Moreover, such improvements in the laser-driven source features will allow reaching better conditions for potential collection and transport of such kind of beams. Indeed, coupled to the investigations recently carried out on different target types, the development of new strategies and advanced techniques for transport, diagnostics, and dosimetry of the optically accelerated particles represents a crucial step toward the clinical use of such non-conventional beams and to achieve well-controlled high-energy beams with suitable and reproducible bunch parameters for medical applications. In this context, a collaboration between the INFN-LNS (Nuclear Physics Laboratory, Catania, Italy) and the ASCR-FZU (Institute of Physics of the Czech Academy of Science) has been established in 2011. The main aim of the collaboration, named ELIMED (ELI-Beamlines MEDical applications), is to demonstrate that high-energy optically accelerated ion beams can be used for multidisciplinary applications, including the hadron therapy case, designing and assembling a complete transport beamline provided with diagnostics and dosimetric sections that will also enable the Users to apply laser-driven ion beams in multidisciplinary fields. In 2012, ELI-Beamlines started the realization of the laser facility, where one of the experimental hall, will be dedicated to ion and proton acceleration and will host the ELIMED beamline. In 2014, a 3-year contract has been signed between INFN-LNS and ELI-Beamlines to develop and realize the ELIMAIA beamline section dedicated to the collection, transport, diagnostics, and dosimetry of laser-driven ion beams. This section, named ELIMED as the collaboration, will be entirely developed by the LNS-INFN and will be delivered and assembled in the ELIMAIA experimental hall within the end of 2017. One of the purposes of the ELIMAIA beamline is to provide to the interested scientific community a user-oriented facility where accurate dosimetric measurements and radiobiology experiments can be performed (48). The technical solution proposed for the realization of the ELIMED beamline are described in Ref. (49). A schematic layout of the ELIMED section along the ELIMAIA beamline is shown in Figure 12.

The beam transport line consists of an in vacuum section dedicated to the collection transport and selection of the optically accelerated particles. In particular, few cm downstream the target, a focusing system based on permanent magnet quadrupoles (PMQs) will be placed. A complete description of the designed system along with the study of the PMQs optics for different energies is given in Ref. (50). The focusing system will be coupled to a selector system (ESS) dedicated to the beam selection in terms of species and energy. The ESS consists of a series of 4 C-shape electromagnet dipoles. The magnetic chicane is based on a fixed reference trajectory with a path length of about 3 m. According to the feasibility study results, such a solution will allow to deliver ions up to 60 MeV/n with an energy bandwidth, depending on the slit aperture, varying from 5% up to 20% at the highest energies and for the different species selected ensuring a rather good transmission efficiency, 10^6^–10^11^ ions/pulse. At the end of the in vacuum beamline, downstream the ESS, a set of conventional electromagnetic transport elements, two quadrupoles and two steering magnets, will allow refocusing of the selected beam and correcting for any possible misalignment. This last transport section will also allow providing a variable beam spot size between 0.1 and 10 mm.

A complete Monte Carlo simulation of the entire beamline and of the associated detectors (51) has been also performed using the Geant4 toolkit (24, 26). Moreover, when the system simulation will be ready, it will be used to study and optimize the particle transport at well-defined positions. The evaluation of dose, fluence, and particle distribution in the in-air section will be performed as well.

According to the beam transport simulation results, performed for the 60-MeV case with the beamline elements designed for ELIMAIA and considering a typical TNSA-like distribution with a cutoff energy of about 120 MeV and an angular divergence with a FWHM of 5 at 60 MeV, it is possible to deliver 60 MeV proton beam with a 20% energy spread with a rather uniform 10 mm × 10 mm spot size, beam divergence less than 0.5° and achieving a transmission efficiency of about 12%.

The simulation studies permitted us to estimate, in the worst conditions for the generated beams (biggest angular spread lowest expected particles number) the dose reaching the end of the beamline at each bunch. The value of 2 cGy per pulse for 60 MeV protons was found. This value, assuming a laser repetition rate of 1 Hz, would provide a pulsed proton beam with an average dose rate of about 1.2 Gy/min, which represents the minimal requirement for typical radiobiology experiments and is also promising considering the future possibility of running the PW laser available at ELI-Beamlines at a repetition rate of 10 Hz. Radiobiology experiments with laser-driven beams need on-line dosimetry measurements with a level of accuracy within 5%. Moreover, precise evaluations of the absolute dose released by the incoming radiation represent an extreme important requirement for many applications, as for instance the hadrontherapy one. However, the very high dose rate and the limited shot-to-shot reproducibility characterizing the laser-driven ion beams, do not allow to easily performing dose measurements, with the required accuracy, using conventional devices. Indeed, several effects have to be considered with high intensity, pulsed ion beams, as gas recombination, dose–rate dependence, and not-negligible electromagnetic pulse. Therefore, since no dosimetry protocol has been established, new technologies and innovative dosimeters must be developed to perform a correct, on-line dose measurement with laser-driven ion beams. The in-air section of the ELIMED beamline dedicated to dosimetry and irradiation will be composed of three main elements: a secondary emission monitor (SEM) and a multi-gap transmission ionization chamber (IC), will be used for relative dose measurements, whereas a Faraday Cup (FC), specifically designed to decrease the uncertainties in the collected charge has been realized (52) and will be placed at the irradiation point for absolute dose measurements. Moreover, a sample irradiation system (SIS) will be installed at the end of the in-air section, allowing the positioning of the cell samples with a sub-millimeter precision.

**Figure 13:**
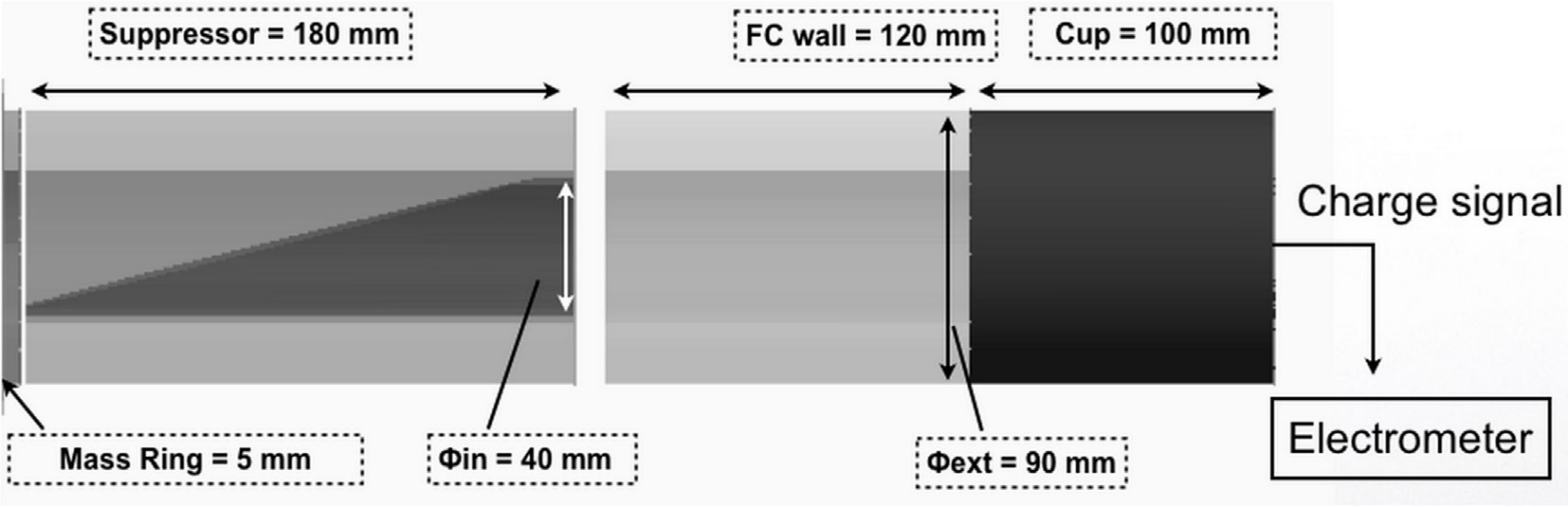
Layout of the ELIMED Faraday Cup. Dimensions of the main components are indicated.

An accurate measurement of the absolute dose using a FC requires a precise measurement of the total charge carried by the beam, of the proton beam energy spectrum, and of the effective beam area; the latter needed to extract the fluence distribution (53). A typical Faraday Cup, used for ion beam dosimetry (53), consists of a thin entrance window, a suppressor electrode aimed to repel secondary electrons, and a collecting cup, able to stop the impinging primary beam and to collect the total charge as shown in Figure 13. In addition, our FC design, inspired by similar detectors already developed for ion beam dosimetry (54), contains a second beveled electrode, coaxial and internal to the standard one, aimed to optimize the charge collection efficiency and reduce the uncertainties, related to the charge collection, caused by the secondary electrons produced, as visible in Figure 13. The beam area and energy spectrum, needed for dose evaluation, will be measured using Gafchromic films (55). These dosimeters, although allow to obtain spatial dose distributions with high spatial resolution, are passive detectors, thus they need a post processing analysis. Further alternative solutions based optical fiber and spectrometer consisting of scintillator stacks to perform, respectively, on-line beam spot and energy spectrum measurements are currently under investigation. The detailed description of the Faraday Cup and the preliminary results obtained using conventional proton beams are discussed in Ref. (49). Other dosimetric systems are under consideration and evaluation. In particular, the use of TLD (Thermoluminescent detector) is under consideration as they are not sensible to the light with an acceptance dose range much extended as respect the CR39 detector. Preliminary tests and calibration procedures, using the TLD800 detectors model, have been already discussed and defined by the medical physicists of the Cannizzaro Hospital in Catania, where a long tradition and big expertise in these detectors is settled. TLD800, in fact, can detect doses in the range of the microgray that are the quantities expected in the first phases of the project.

## 6 Conclusion and Future Perspectives

CATANA is the first Italian proton therapy facility where 62 MeV protons have been used for the radiotherapy treatment of ocular melanomas. Since 2002, about 294 patients have been treated and follow-up results are consistent with the statistics so far produced (see PTCOG web site: http://www.ptcog.ch/). Many research studies have been triggered by the proton therapy activities. Among these, the development of new detectors and quality assurance methodology are of particular interest.

Moreover, the idea of new irradiation approaches, based on the use of laser-accelerated beams, has been developed. The latter was possible thanks to the collaboration with the ELI-Beamlines facility, where a new, users-open transport beamline for laser-accelerated beams will be realized and installed by INFN.

## Author Contributions

GC is the main proposer of the CATANA activity. GAPC and DM are the main proposers of the ELIMED activity and GAPC is responsible of the CATANA proton therapy room. GAPC, GPetringa, and FR contributed on the relative dosimetry and on the Monte Carlo simulations. FR and VScuderi contributed in the experimental and dosimetric part of the paper with particular regard to the laser-driven activities. FS and AntonioR are the main responsible of the ELIMED transport beamline. VSalamone and LR contributions are on absolute dosimetry, dosimetry tests, and patients positioning. They are the medical physicists following the treatments. CS and GPrivitera are the oncologists and radiotherapist dedicated to the treatments. TA and AndreaR are the oculists who follow the patients after the treatment producing the follow-up results. VP, MS, and LV are the medical physicists involved in the use of TLD detectors in the laser-driven proton beams. GL, RL and AA contributed to the ELIMED dosimetry working on the Faraday Cup tests. GM contributed on the diagnostic and, partially, on the Monte Carlo activities of ELIMED.

## Conflict of Interest Statement

The authors declare that the research was conducted in the absence of any commercial or financial relationships that could be construed as a potential conflict of interest.
