# Corrigendum: Clinical and Research Activities at the CATANA Facility of INFN-LNS: From the Conventional Hadrontherapy to the Laser-Driven Approach

**DOI:** 10.3389/fonc.2017.00247

**Published:** 2017-10-31

**Authors:** Giuseppe A. P. Cirrone, Giacomo Cuttone, Luigi Raffaele, Vincenzo Salamone, Teresio Avitabile, Giuseppe Privitera, Corrado Spatola, Antonio G. Amico, Giuseppina Larosa, Renata Leanza, Daniele Margarone, Giuliana Milluzzo, Valeria Patti, Giada Petringa, Francesco Romano, Andrea Russo, Antonio Russo, Maria G. Sabini, Francesco Schillaci, Valentina Scuderi, Lucia M. Valastro

**Affiliations:** ^1^Laboratori Nazionali del Sud, Istituto Nazionale di Fisica Nucleare (INFN-LNS), Catania, Italy; ^2^Azienda Ospedaliero Universitaria Policlinico Vittorio Emanuele, Presidio Gaspare Rodolico, Catania, Italy; ^3^ELI-Beamlines Project, Institute of Physics ASCR, v.v.i. (FZU), Prague, Czechia; ^4^Medical Physics Section, Cannizzaro Hospital, Catania, Italy; ^5^National Physical Laboratory, Acoustic and Ionizing Radiation Division, Middlesex, United Kingdom

**Keywords:** protontherapy, dosimetry, clinical follow-up, Monte Carlo, laser-driven, ELIMED

Due to a mistake, Dr. A. G. Amico, Dr. G. Larosa, Dr. R. Leanza, and Dr. G. Milluzzo were not included as authors in the published article. The authors apologize for this error and state that this does not change the scientific conclusions of the article in any way.

The corrected Author Contributions appears below.

GC is the main proposer of the CATANA activity. GAPC and DM are the main proposers of the ELIMED activity and GAPC is responsible of the CATANA proton therapy room. GAPC, GPetringa, and FR contributed on the relative dosimetry and on the Monte Carlo simulations. FR and VScuderi contributed in the experimental and dosimetric part of the paper with particular regard to the laser-driven activities. FS and AntonioR are the main responsible of the ELIMED transport beamline. VSalamone and LR contributions are on absolute dosimetry, dosimetry tests, and patients positioning. They are the medical physicists following the treatments. CS and GPrivitera are the oncologists and radiotherapist dedicated to the treatments. TA and AndreaR are the oculists who follow the patients after the treatment producing the follow-up results. VP, MS, and LV are the medical physicists involved in the use of TLD detectors in the laser-driven proton beams. GL, RL and AA contributed to the ELIMED dosimetry working on the Faraday Cup tests. GM contributed on the diagnostic and, partially, on the Monte Carlo activities of ELIMED.

The original article was also updated.

## Conflict of Interest Statement

The authors declare that the research was conducted in the absence of any commercial or financial relationships that could be construed as a potential conflict of interest.

